# Hemoglobin-to-Red cell distribution width ratio and asthma risk: cross-population validation and vitamin D mediation analysis

**DOI:** 10.3389/falgy.2026.1819644

**Published:** 2026-06-05

**Authors:** Yanbiao Xiao, Jun Lai, Yanglin Zhang

**Affiliations:** 1 Department of Plastic Surgery, Ganzhou Dermatosis Hospital, Ganzhou, Jiangxi Province, China; 2 Department of Pharmacy, Ganzhou People’s Hospital, Ganzhou, Jiangxi Province, China

**Keywords:** asthma, external validation, HRR, mediation analysis, NHANES, vitamin D

## Abstract

**Purpose:**

This study aimed to explore the association between hemoglobin-to-red blood cell distribution width ratio (HRR) and asthma risk using a large U.S. population-based cohort (NHANES), and to validate the core association in an independent Chinese clinical cohort (Ganzhou People's Hospital) to improve cross-population generalizability. The mediating role of vitamin D in the HRR-asthma association was further explored only in the NHANES discovery cohort.

**Patients and methods:**

Data from NHANES 2011–2018 and 2021–2023 (*n* = 19,140) were used as the discovery cohort. An independent clinical cohort from Ganzhou People's Hospital (*n* = 519) was used for external validation. Logistic regression models were constructed to assess the HRR-asthma association with stepwise adjustment for confounders. Variance inflation factor (VIF) was used to examine multicollinearity. Discrimination (AUC), calibration (Hosmer–Lemeshow test), and clinical utility (DCA) were evaluated. Mediation analysis was only performed in the NHANES cohort due to unavailability of vitamin D data in the clinical cohort.

**Results:**

In the NHANES cohort, fully adjusted logistic regression showed a significant inverse association between HRR and asthma (OR = 0.60, 95%CI: 0.45–0.80, *P* < 0.001), and vitamin D completely mediated this association (indirect effect *β*=0.011, *P* = 0.001). In the Ganzhou cohort, the inverse association was consistently validated (fully adjusted OR = 0.21, 95%CI: 0.06–0.75, *P* = 0.017). The HRR-integrated multivariable model showed moderate discrimination in the NHANES cohort (AUC=0.539, 95%CI: 0.528–0.551) with good calibration (*P* = 0.32). In the Ganzhou cohort, the multivariable prediction model exhibited moderate discriminatory ability (AUC=0.718, 95%CI: 0.667–0.770), whereas HRR alone showed low-to-moderate discrimination (AUC=0.375, equivalent to 0.625 for the inverse association).

**Conclusion:**

HRR is inversely associated with asthma risk across U.S. general and Chinese clinical populations. Vitamin D fully mediates the association in NHANES but could not be validated in the clinical cohort. HRR is a simple, low-cost, cross-population risk-associated component within a comprehensive clinical prediction model, rather than a standalone diagnostic marker.

## 1 Introduction

Asthma, a prevalent chronic inflammatory airway disease, imposes a substantial global health burden. Recent data from the Global Burden of Disease study estimated 262 million prevalent cases worldwide in 2019 (1). Identifying reliable, accessible biomarkers for asthma risk assessment is crucial for early intervention and prognosis improvement.

Hemoglobin-to-red blood cell distribution width ratio (HRR) is a novel inflammation-related biomarker that has gained increasing attention in medical research (2–5). HRR integrates both hemoglobin (Hb) and Red blood cell distribution width (RDW), reflecting erythrocyte maturity, heterogeneity, and inflammatory status simultaneously, making it more stable than single markers in inflammatory diseases. HRR reflects key erythrocyte characteristics. Erythrocytes not only participate in oxygen transport (6) but also play critical roles in immune regulation and inflammatory responses (7). In patients with asthma, prolonged airway inflammation and hypoxia may interfere with erythrocyte metabolism and production, leading to changes in HRR.

A recent population-based study using NHANES data confirmed an inverse association between HRR and asthma risk and asthma exacerbations (8). However, that study was limited to a single U.S. cohort without external validation across ethnicities, and no prior study has explored mediating mechanisms underlying the HRR-asthma relationship. Thus, the generalizability and mechanistic basis of this association remain unclear.

Public database studies (e.g.,NHANES) provide large sample sizes and broad population representativeness, but their generalizability to distinct ethnicities or clinical settings is often questioned (9). External validation using independent cohorts, especially clinical cohorts with direct patient care data, is increasingly mandated by journals to confirm the robustness of associations (10). Vitamin D is a key regulator of immune function and inflammation (11, 12), but its role in mediating the HRR-asthma association has not been validated across diverse populations.

To address these critical gaps, we used NHANES 2011–2018 and 2021–2023 as a discovery cohort and an independent Chinese clinical cohort (Ganzhou People's Hospital) for external validation. This dual-cohort design enhances reliability and meets modern publication standards for public database research.

The present study aimed to:

1. Investigate the association between HRR and asthma risk;
2. Validate the stability and generalizability of this association across populations;
3. Explore the potential mediating effect of vitamin D in the NHANES cohort.

## 2 Materials and methods

### 2.1 Study cohorts

#### 2.1.2 Discovery cohort: NHANES 2011–2018 and 2021–2023

The NHANES uses a stratified multistage probability sampling design to represent the U.S. population. A total of 19140 participants were included after excluding those with missing data on HRR, asthma, vitamin D, or key covariates. The study was approved by the National Center for Health Statistics (NCHS) Ethics Review Committee, and all participants provided informed consent (13). Given the potential disruptions in sampling procedures and the unavailability of key laboratory variables (Hb and RDW) during the pandemic, NHANES cycles from 2019 to 2021 were excluded from the final analysis.

### 2.2 External validation cohort: ganzhou people's hospital

A retrospective cross-sectional clinical cohort was established at Ganzhou People's Hospital in 2025. A total of 519 eligible participants were retrospectively identified and enrolled by reviewing electronic medical records. Eligibility criteria: Inclusion Criteria: (1) Available complete blood count results for calculation of HRR (Hb [g/dL]/RDW [%]); (2) Confirmed asthma diagnosis(based on clinical symptoms, lung function tests, and physician diagnosis) or no asthma (ruled out by clinical evaluation); (3) No missing key covariates (age, gender, BMI, smoking status, drinking, hypertension, diabetes). Exclusion Criteria: (1) Severe hematological disorders that may affect hemoglobin or RDW levels; (2) Acute severe infection, active bleeding, or critical organ dysfunction; (3) Other chronic respiratory diseases (e.g., COPD, interstitial lung disease) that may confound asthma diagnosis; (4) Incomplete clinical or laboratory data.Participants were retrospectively identified from hospitalized patients using electronic medical records.

Asthma diagnosis was based on physician diagnosis, clinical symptoms, and lung function tests, consistent with the Global Initiative for Asthma (GINA) 2024 guidelines (14). Non-asthma participants were defined as individuals with no clinical or laboratory evidence of asthma or other chronic airway diseases after full review. The study was approved by the Ethics Committee of Ganzhou People's Hospital, and written informed consent was obtained from all participants.

## 3 Variable definitions

### 3.1 Outcome Variable: asthma

NHANES: Defined as a positive response to “Has a doctor or other health professional told you that you have asthma?” (15, 16).

Ganzhou People’s Hospital: Defined as clinical diagnosis of asthma (consistent with the Global Initiative for Asthma (GINA) 2024 criteria)with documented symptoms (wheezing, dyspnea).

### 3.2 Exposure Variable: HRR

Calculated as Hb (g/dL) divided by RDW (%),using laboratory data from each cohort (17). HRR was stratified into quartiles for subgroup analyses. Both cohorts used automated hematology analyzers with standardized routine quality control procedures. Although different reagents were used across laboratories, all Hb and RDW measurements were calibrated against international reference standards, ensuring high comparability between the two cohorts.

### 3.3 Mediator: vitamin D

NHANES: Serum total vitamin D (nmol/L) = 25-hydroxyvitamin D2 + 25-hydroxyvitamin D3 (18).

Ganzhou People’s Hospital: Vitamin D data were not available due to clinical practice limitations; this cohort focused on validating the HRR-asthma association (the core exposure-outcome relationship).

### 3.4 Covariates

Lifestyle factors include smoking and alcohol consumption status. Disease states include hypertension and diabetes mellitus, which, as common comorbidities,significantly increase the risk of developing Asthma (19–21). Consistent covariates adjusted in both cohorts:age,gender,marital status,smoking status (yes/no), drinking status (yes/no), hypertension (yes/no), diabetes (yes/no), and BMI(kg/m^2^). Additional covariates in NHANES: race and education level (adapted to U.S.population characteristics).

## 4 Statistical analysis

Descriptive statistics were performed for all variables. Continuous variables were presented as mean ± standard deviation (SD) or median (interquartile range, IQR, Q1–Q3). Categorical variables were expressed as frequencies and percentages. Between-group comparisons were performed using independent t-tests or one-way ANOVA for continuous variables, and chi-square tests for categorical variables. Variance inflation factor (VIF) was used to evaluate multicollinearity among covariates. All VIF values were less than 2, indicating no significant multicollinearity. Interaction tests between HRR and age group (<60 vs. ≥ 60 years) and between HRR and gender were conducted in both cohorts to examine effect modification. In the NHANES cohort, a significant interaction was observed between HRR and age (*P* for interaction = 0.023), whereas no significant interaction was detected for HRR and gender (*P* = 0.452). In the Ganzhou cohort, no significant interactions were observed for either HRR × age (*P* = 0.477) or HRR × gender (*P* = 0.545).

Multivariable logistic regression models were constructed to explore the association between HRR and asthma risk. Four nested models were applied in both cohorts:

- Model 1: Unadjusted
- Model 2: Adjusted for age and gender
- Model 3: Further adjusted for marital status, smoking status, and drinking status
- Model 4: Fully adjusted model, additionally adjusted for BMI, hypertension, and diabetes.

All models were adjusted for key confounders that may influence erythrocyte indices, systemic inflammation, or asthma risk, including age, gender, smoking status, BMI, hypertension, and diabetes.

In the external validation cohort, model performance was evaluated using three metrics: Discrimination: Receiver operating characteristic (ROC) curve and area under the curve (AUC). Calibration: Calibration curve and Hosmer-Lemeshow test (*P* > 0.05 indicated good calibration). Decision curve analysis (DCA) to compare the net benefit of the HRR-integrated model with “treat none” or “treat all” strategies.

Subgroup analyses stratified by age (<60 vs. ≥ 60 years) and gender were performed in both cohorts. Mediation analysis (three-pathway method) was conducted only in the NHANES cohort to evaluate the mediating effect of vitamin D, because vitamin D data were unavailable in the clinical cohort.

All statistical analyses were performed using SPSS 27.0 and R software (version 4.2.3). A two-sided *P* < 0.05 was considered statistically significant.

## 5 Results

### 5.1 Baseline characteristics of the Two cohorts

#### 5.1.1 NHANES cohort (*n* = 19140)

Among 19,140 participants, 3,071 (16.04%) had asthma. Asthma patients were younger (47.75 ± 17.38 vs. 50.33 ± 17.33 years), more likely to be female (56.76% vs. 47.16%), and had higher BMI (30.78 ± 7.95 vs. 29.11 ± 6.72 kg/m^2^) and RDW (13.73 ± 1.39 vs. 13.61 ± 1.32%) than non-asthma participants (all *P* < 0.001). HRR was significantly lower in the asthma group (1.03 ± 0.16 vs. 1.05 ± 0.16, *P* < 0.001) (Supplementary Table S1).

#### 5.1.2 Ganzhou cohort (*n* = 519)

Of 519 patients, 101 (19.46%) had asthma. Compared with non-asthma patients, asthma patients were older (64.00 vs. 61.00 years), more likely to be female (49.50% vs. 34.93%), and had lower Hb (12.30[11.30,13.40] vs. 13.05[11.72,14.30]g/dL) and HRR (0.93[0.77,1.04] vs. 1.00[0.88,1.12]) (all *P* < 0.05) (Supplementary Table S2).

### 5.2 HRR and asthma risk in the NHANES cohort and ganzhou cohort

In fully adjusted Model 4, HRR was negatively associated with asthma (OR = 0.60,95%CI:0.45–0.80,*P* < 0.001). When HRR was stratified into quartiles, the highest quartile(Q4) had a 22%lower asthma risk than the lowest quartile (Q1) (OR = 0.76,95%CI:0.66–0.86,*P* < 0.001) (Supplementary Table S3). The negative association between HRR and asthma was consistently validated in the Ganzhou People's Hospital Cohort. In fully adjusted Model 4, each unit increase in HRR was associated with a 79%reduction in asthma risk (OR = 0.21,95%CI:0.06–0.75,*P* = 0.017). Quartile analysis showed Q4 vs. Q1 had an OR of 0.45 (95%CI:0.22–0.95,*P* = 0.037)(Supplementary Table S4). Consistent with the discovery cohort, significant inverse trends between HRR quartiles and asthma risk were observed in the clinical cohort (Ptrend < 0.05). Given the relatively small sample size, quartile stratification was used for consistency with the NHANES analysis, whereas continuous HRR showed a more robust and stable dose–response association.

### 5.3 Discriminatory performance of the HRR-integrated model and HRR alone

**Figure 1:**
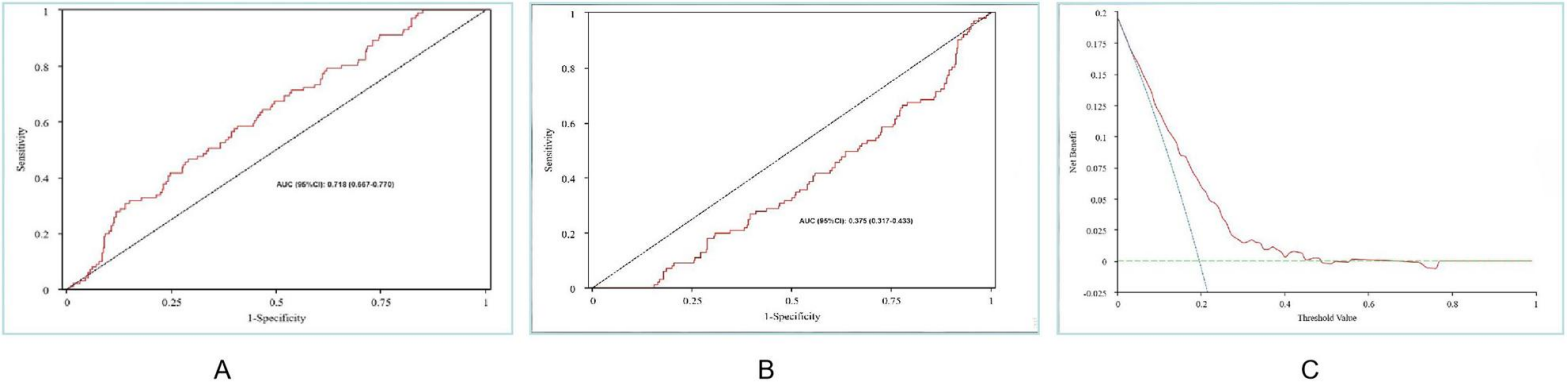
(A, B) Receiver operating characteristic (ROC) curves of HRR for predicting asthma risk in the training and validation cohorts. (C) Decision curve analysis of the HRR model, showing the net benefit across different threshold probabilities.

In the Ganzhou validation cohort, the discriminatory performance of the HRR-integrated multivariable model and HRR as a standalone biomarker were evaluated separately (Figure 1). The multivariable prediction model, which incorporated HRR and key clinical covariates including age, gender, smoking status, BMI, hypertension, and diabetes, demonstrated moderate discriminatory ability for asthma risk stratification, with an AUC of 0.718 (95% CI: 0.667–0.770, *P* < 0.001; Figure 1A).

As a single marker, HRR showed low-to-moderate discriminatory performance, with an AUC of 0.375 (95% CI: 0.318–0.432, *P* < 0.001; Figure 1B). The AUC value below 0.5 was expected given the significant inverse association between HRR and asthma risk, and is statistically equivalent to an AUC of 0.625 for a positive predictive relationship.

The Hosmer-Lemeshow test indicated excellent calibration (*P* = 0.981), confirming good agreement between the model-predicted asthma risk and the actual observed asthma incidence. Decision curve analysis (DCA) revealed that the multivariable model yielded mild positive net benefit at threshold probabilities below 0.2, while net benefit was negligible at higher thresholds (Figure 1C).

**Figure 2:**
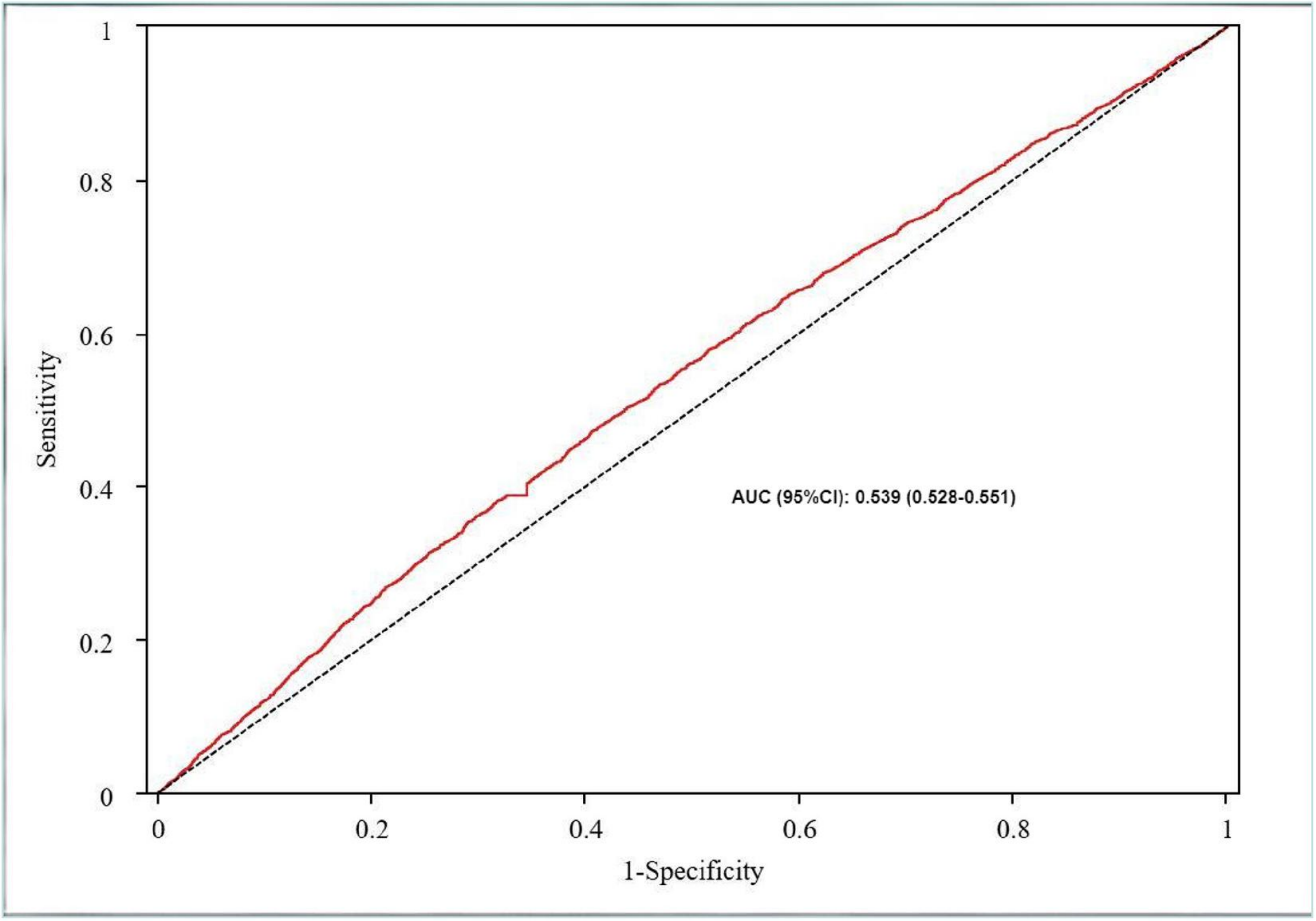
Receiver operating characteristic (ROC) curve of HRR for predicting asthma risk in the external validation cohort.

In the NHANES discovery cohort, the HRR-integrated multivariable model also showed moderate discrimination (AUC=0.539, 95%CI: 0.528–0.551) with good calibration (*P* = 0.32) (Figure 2).

Collectively, these findings support HRR as a useful component in multivariable risk stratification models rather than a standalone diagnostic tool for asthma.

### 5.4 Subgroup and interaction analyses

Interaction effects between HRR and age group, as well as HRR and gender, were tested to evaluate potential effect modification. In the NHANES discovery cohort, a significant interaction was observed between HRR and age group (*P* for interaction = 0.023), while no significant interaction was detected between HRR and gender (*P* for interaction = 0.452). Consistent with the interaction analysis, subgroup analyses revealed that the inverse association between HRR and asthma risk was only significant in older adults (OR = 0.58, 95%CI: 0.35–0.97, *P* = 0.039) compared with younger adults (OR = 0.72, 95%CI: 0.51–1.02, *P* = 0.061) in the NHANES cohort.

In the Ganzhou external validation cohort, no significant interactions were observed for either HRR × age (*P* = 0.477) or HRR × gender (*P* = 0.545). Subgroup analyses confirmed the robustness of the HRR–asthma association in older adults (OR = 0.15, 95%CI: 0.03–0.85, *P* = 0.032), while the association in younger adults did not reach statistical significance (all *P* > 0.05) (Supplementary Table S5).

### 5.5 Mediating role of vitamin D (NHANES cohort)

Mediation analysis showed vitamin D fully mediated the HRR-asthma association: Total effect of HRR on asthma: *β*=−0.015(*P* = 0.44); Indirect effect via vitamin D: *β*=0.011(*P* = 0.001); Direct effect:*β*=−0.026(*P* = 0.185); Mediation propo-rtion:100%(Supplementary Table S6).

## 6 Discussion

This study combined a large U.S. population-based cohort (NHANES) and an independent retrospective Chinese clinical cohort to investigate the association between the hemoglobin-to-red cell distribution width ratio (HRR) and asthma risk. The consistent inverse association observed across ethnically, geographically, and clinically distinct populations strengthens the reliability of HRR as a potential inflammatory-related biomarker for asthma risk assessment.

Hemoglobin and red blood cell distribution width (RDW) are core components of the complete blood count (CBC), a ubiquitous, low-cost, and widely accessible laboratory test in clinical practice. Both indices have been extensively linked to systemic inflammation, hypoxia, and adverse clinical outcomes across multiple disease domains. In respiratory diseases, elevated RDW and reduced hemoglobin have been consistently associated with poor lung function, increased risk of chronic obstructive pulmonary disease (COPD) exacerbations, and adverse prognosis in acute respiratory failure. Notably, a recent population-based study using NHANES data demonstrated that higher HRR was significantly associated with better pulmonary function (FVC, FEV1, PEF, and FEF 25%–75%) in middle-aged and older adults, further supporting HRR as a promising biomarker for lung health (22). As a composite biomarker integrating hemoglobin and RDW, HRR has emerged as a more stable and comprehensive indicator of systemic inflammation and erythropoietic status than either marker alone. HRR has been validated as a robust prognostic predictor in diverse clinical contexts, including some cancers, ischemic stroke, and diabetes-related cardiovascular complications, demonstrating its broad utility as a systemic inflammatory biomarker (23–25).

Despite this growing body of evidence, research on HRR in asthma remains extremely limited. To date, only one recent population-based study (8) has reported an inverse association between HRR and asthma risk in the U.S. NHANES cohort. However, that study was restricted to a single ethnic population, lacked independent external validation, and did not explore underlying mechanistic pathways.

The present study addresses these critical gaps by: (1) confirming the inverse association between HRR and asthma risk in a large, nationally representative U.S. cohort; (2) validating these findings in an independent Chinese clinical cohort to support cross-ethnic and cross-setting generalizability; and (3) identifying vitamin D as a full mediator of the HRR–asthma relationship in the population-based cohort, providing a mechanistic foundation for the observed association. This dual-cohort design, combined with mediation analysis, significantly advances the understanding of HRR as a potential biomarker for asthma risk assessment.

Mechanistic insight: Vitamin D fully mediates the HRR-asthma association in the NHANES cohort, consistent with previous evidence that vitamin D regulates immune function and erythropoiesis (26–28). This suggests HRR may influence asthma risk by modulating vitamin D levels, providing a theoretical basis for vitamin D supplementation in high-risk individuals (e.g.,low HRR).

The magnitude of the HRR-asthma association was stronger in the validation cohort (OR = 0.21 vs. 0.65 in NHANES), which may be attributed to: Population differences: NHANES includes healthy individuals and mild asthma cases,while the clinical cohort comprises patients with more severe asthma (higher disease burden),amplifying the HRR effect; Diagnostic criteria: Asthma in the clinical cohort was confirmed by objective tests (lung function),reducing misclassification bias compared to NHANES’s self-reported diagnosis; Ethnic and lifestyle differences: Chinese populations may have distinct vitamin D metabolism or inflammatory profiles, which could modify the HRR-asthma association.

Subgroup analyses reinforced a more prominent protective association among individuals aged 60 years and older in both cohorts, consistent with the significant interaction detected in NHANES. Older adults commonly experience age-related changes in erythropoiesis, higher prevalence of vitamin D insufficiency, and greater systemic inflammation, all of which may amplify the influence of HRR on asthma risk. The relative stability of the association across gender groups further supports the potential utility of HRR as a broadly applicable risk marker rather than one limited to specific demographic subgroups.

Interpretation of AUC values requires careful distinction between risk stratification and diagnostic testing. In the Ganzhou cohort, HRR alone yielded an AUC of 0.375, which, given the inverse association, is statistically equivalent to an AUC of 0.625 for a positive predictor. This indicates that HRR has limited standalone diagnostic accuracy for identifying individual asthma patients.

However, when incorporated into a multivariable clinical model, the AUC improved to 0.718, supporting its value as a contributing component of risk stratification. HRR is best interpreted as a population-level screening tool rather than a stand-alone diagnostic biomarker. Its clinical relevance lies in identifying subgroups at elevated risk (e.g., individuals with low HRR) who may benefit from further evaluation, rather than replacing formal pulmonary function testing or standardized clinical assessment.

Several limitations should be acknowledged. First, the mediating effect of vitamin D was only examined in the NHANES cohort and could not be validated in the Chinese clinical cohort due to unavailability of vitamin D measurements, which restricts the generalizability of our mechanistic inferences. Second, the single-center design and relatively small sample size of the validation cohort may limit external validity and increase the potential for selection bias. Third, ethnic, lifestyle, and healthcare system differences between the two populations may further affect the cross-population generalizability of our results. Finally, despite adjustment for major comorbidities and lifestyle factors, residual confounding cannot be excluded, particularly from unmeasured factors such as specific nutritional deficiencies, anemia subtypes, and detailed inflammatory markers.

Future prospective studies with larger, multi-center, multi-ethnic designs are warranted to confirm the causal nature and generalizability of our findings. Further interventional studies investigating vitamin D supplementation among individuals with low HRR may also help clarify the clinical potential of HRR-guided strategies for asthma prevention. These limitations should be taken into consideration when interpreting the observed association between HRR and asthma.

## Conclusion

HRR is inversely associated with asthma risk in both a U.S. population-based cohort and a Chinese clinical cohort, with consistent findings across key age and gender subgroups. Vitamin D may fully mediate this association in the general population. Although its standalone discriminatory performance is modest, HRR demonstrates potential as a low-cost, readily accessible biomarker for population-level asthma risk stratification and clinical management. Further prospective and multi-center studies are needed to confirm causal relationships, refine integrated risk prediction models, and explore the value of HRR-guided strategies including vitamin D supplementation for asthma prevention.

## Data Availability

The original contributions presented in the study are included in the article/Supplementary Material, further inquiries can be directed to the corresponding author.
