# Supplementary material for: Hemoglobin-to-Red cell distribution width ratio and asthma risk: cross-population validation and vitamin D mediation analysis
Source: Front Allergy. 2026 Jun 5;7:1819644. doi: 10.3389/falgy.2026.1819644 (PMC13279617; doi:10.3389/falgy.2026.1819644)
Supplement: Supplementary file 1 [file Table1.docx]

Table 1 Baseline Characteristics by Asthma Status(NHANES 2011–2023)

| Variables | Total (n = 19140) | No Asthma(n = 16069) | Asthma (n = 3071) | *P* |
| --- | --- | --- | --- | --- |
|  |  |  |  |  |
| Age, years | 49.92 ± 17.37 | 50.33 ± 17.33 | 47.75 ± 17.38 | <.001 |
| BMI, kg/m^2^ | 29.38 ± 6.96 | 29.11 ± 6.72 | 30.78 ± 7.95 | <.001 |
| WC, cm | 100.25 ± 16.72 | 99.78 ± 16.38 | 102.72 ± 18.22 | <.001 |
| VitaminD, nmol/L | 69.81 ± 31.97 | 69.59 ± 31.59 | 70.96 ± 33.83 | 0.037 |
| TC, mg/dL | 190.26 ± 41.98 | 190.71 ± 41.80 | 187.93 ± 42.80 | <.001 |
| Hb, g/dL | 14.05 ± 1.50 | 14.08 ± 1.50 | 13.91 ± 1.51 | <.001 |
| RDW, % | 13.63 ± 1.33 | 13.61 ± 1.32 | 13.73 ± 1.39 | <.001 |
| HRR | 1.04 ± 0.16 | 1.05 ± 0.16 | 1.03 ± 0.16 | <.001 |
| Gender, n(%) |  |  |  | <.001 |
| Male | 9819 (51.30) | 8491 (52.84) | 1328 (43.24) |  |
| Female | 9321 (48.70) | 7578 (47.16) | 1743 (56.76) |  |
| Race, n(%) |  |  |  | <.001 |
| Mexican American | 2251 (11.76) | 2014 (12.53) | 237 (7.72) |  |
| Other | 4386 (22.92) | 3694 (22.99) | 692 (22.53) |  |
| Non-Hispanic White | 8784 (45.89) | 7298 (45.42) | 1486 (48.39) |  |
| Non-Hispanic Black | 3719 (19.43) | 3063 (19.06) | 656 (21.36) |  |
| Education, n(%) |  |  |  | <.001 |
| High school or below | 3241 (16.93) | 2784 (17.33) | 457 (14.88) |  |
| Some college or above | 15899 (83.07) | 13285 (82.67) | 2614 (85.12) |  |
| Maritalstatus, n(%) |  |  |  | <.001 |
| With Partner | 11163 (58.32) | 9543 (59.39) | 1620 (52.75) |  |
| No Partner | 7977 (41.68) | 6526 (40.61) | 1451 (47.25) |  |
| Drinking, n(%) |  |  |  | 0.026 |
| Yes | 3215 (16.80) | 2657 (16.53) | 558 (18.17) |  |
| No | 15925 (83.20) | 13412 (83.47) | 2513 (81.83) |  |
| Smoking, n(%) |  |  |  | <.001 |
| Yes | 8957 (46.80) | 7435 (46.27) | 1522 (49.56) |  |
| No | 10183 (53.20) | 8634 (53.73) | 1549 (50.44) |  |

Table 1 Continued Baseline Characteristics by Asthma Status(NHANES 2011–2023)

| Variables | Total (n = 19140) | No Asthma(n = 16069) | Asthma (n = 3071) | *P* |
| --- | --- | --- | --- | --- |
| Diabetes, n(%) |  |  |  | <.001 |
| Yes | 2610 (13.64) | 2120 (13.19) | 490 (15.96) |  |
| No | 16530 (86.36) | 13949 (86.81) | 2581 (84.04) |  |
| Hypertension, n(%) |  |  |  | <.001 |
| Yes | 6872 (35.90) | 5621 (34.98) | 1251 (40.74) |  |
| No | 12268 (64.10) | 10448 (65.02) | 1820 (59.26) |  |

Note: Continuous variables are presented as mean ± standard deviation (SD); categorical variables as n (%).

Abbreviations: BMI, body mass index; WC, waist circumference; TC, total cholesterol; Hb, hemoglobin; RDW, red blood cell distribution width; HRR, hemoglobin-to-RDW ratio.

Table 2 Baseline Characteristics by Asthma Status (Ganzhou Cohort)

| Variables | Total (n = 519) | No Asthma (n = 418) | Asthma (n = 101) | P |
| --- | --- | --- | --- | --- |
| Age, years | 61.00 (52.00, 71.50) | 61.00 (51.00, 71.00) | 64.00 (58.00, 73.00) | 0.005 |
| BMI, kg/m^2^ | 22.27 (19.90, 24.92) | 22.31 (19.92, 24.94) | 21.88 (19.70, 24.44) | 0.297 |
| Hb, g/dL | 12.90 (11.70, 14.20) | 13.05 (11.72, 14.30) | 12.30 (11.30, 13.40) | <.001 |
| RDW, % | 13.10 (12.60, 13.90) | 13.00 (12.53, 13.80) | 13.40 (12.70, 14.50) | 0.006 |
| HRR | 0.98 (0.86, 1.10) | 1.00 (0.88, 1.12) | 0.93 (0.77, 1.04) | <.001 |
| Maritalstatus, n(%) |  |  |  | 0.537 |
| No Partner | 53 (10.21) | 41 (9.81) | 12 (11.88) |  |
| With Partner | 466 (89.79) | 377 (90.19) | 89 (88.12) |  |
| Drinking, n(%) |  |  |  | 0.536 |
| No | 399 (76.88) | 319 (76.32) | 80 (79.21) |  |
| Yes | 120 (23.12) | 99 (23.68) | 21 (20.79) |  |
| Smoking, n(%) |  |  |  | 0.117 |
| No | 318 (61.27) | 263 (62.92) | 55 (54.46) |  |
| Yes | 201 (38.73) | 155 (37.08) | 46 (45.54) |  |
| Diabetes, n(%) |  |  |  | 0.029 |
| No | 483 (93.06) | 384 (91.87) | 99 (98.02) |  |
| Yes | 36 (6.94) | 34 (8.13) | 2 (1.98) |  |
| Hypertension, n(%) |  |  |  | 0.124 |
| No | 414 (79.77) | 339 (81.10) | 75 (74.26) |  |
| Yes | 105 (20.23) | 79 (18.90) | 26 (25.74) |  |
| Gender, n(%) |  |  |  | 0.007 |
| Male | 323 (62.24) | 272 (65.07) | 51 (50.50) |  |
| Female | 196 (37.76) | 146 (34.93) | 50 (49.50) |  |

Note: Continuous variables are presented as median (Q1, Q3); categorical variables as n (%).

Abbreviations: BMI, body mass index; Hb, hemoglobin; RDW, red blood cell distribution width; HRR, hemoglobin-to-RDW ratio.

Table 3 Association Between HRR and Asthma Risk (NHANES Cohort)

| Variables | Model1 | | Model2 | | Model3 | | Model4 | |
| --- | --- | --- | --- | --- | --- | --- | --- | --- |
|  | OR (95%CI) | *P* | OR (95%CI) | *P* | OR (95%CI) | *P* | OR (95%CI) | *P* |
| HRR(continuous) | 0.47 (0.37 ~ 0.60) | **<.001** | 0.61 (0.46 ~ 0.81) | **<.001** | 0.61 (0.46 ~ 0.81) | **<.001** | 0.60 (0.45 ~ 0.80) | **<.001** |
| HRR quartiles |  |  |  |  |  |  |  |  |
| Q1 (Reference) | 1.00 |  | 1.00 |  | 1.00 |  | 1.00 |  |
| Q2 | 0.82 (0.74 ~ 0.92) | **<.001** | 0.84 (0.75 ~ 0.94) | **0.002** | 0.85 (0.76 ~ 0.94) | **0.003** | 0.84 (0.77 ~ 0.96) | **0.002** |
| Q3 | 0.73 (0.65 ~ 0.81) | **<.001** | 0.77 (0.68 ~ 0.86) | **<.001** | 0.77 (0.69 ~ 0.87) | **<.001** | 0.77 (0.70 ~ 0.89) | **<.001** |
| Q4 | 0.69 (0.62 ~ 0.77) | **<.001** | 0.76 (0.67 ~ 0.87) | **<.001** | 0.76 (0.67 ~ 0.87) | **<.001** | 0.76 (0.68 ~ 0.89) | **<.001** |

Notes: Model 1: unadjusted; Model 2: adjusted for sex, age, race; Model 3: additionally adjusted for education, marital status; Model 4: fully adjusted.

Abbreviations: OR, odds ratio; CI, confidence interval; HRR, hemoglobin-to-RDW ratio.

Table 4 Association Between HRR and Asthma Risk (Ganzhou Cohort)

| Variables | Model1 | |  | Model2 | |  | Model3 | |  | Model4 | |
| --- | --- | --- | --- | --- | --- | --- | --- | --- | --- | --- | --- |
|  | OR (95%CI) | *P* |  | OR (95%CI) | *P* |  | OR (95%CI) | *P* |  | OR (95%CI) | *P* |
| HRR(continuous) | 0.13 (0.04 ~ 0.41) | <.001 |  | 0.22 (0.07 ~ 0.74) | 0.014 |  | 0.27 (0.08 ~ 0.93) | 0.039 |  | 0.21 (0.06 ~ 0.75) | 0.017 |
| HRR quartiles |  |  |  |  |  |  |  |  |  |  |  |
| Q1 (Reference) | 1.00 |  |  | 1.00 |  |  | 1.00 |  |  | 1.00 |  |
| Q2 | 0.80 (0.46 ~ 1.42) | 0.449 |  | 0.74 (0.42 ~ 1.33) | 0.313 |  | 0.74 (0.41 ~ 1.36) | 0.332 |  | 0.70 (0.38 ~ 1.30) | 0.261 |
| Q3 | 0.60 (0.33 ~ 1.08) | 0.086 |  | 0.68 (0.37 ~ 1.24) | 0.206 |  | 0.70 (0.38 ~ 1.29) | 0.254 |  | 0.63 (0.34 ~ 1.18) | 0.147 |
| Q4 | 0.30 (0.15 ~ 0.61) | <.001 |  | 0.44 (0.21 ~ 0.90) | 0.025 |  | 0.47 (0.23 ~ 0.99) | 0.047 |  | 0.45 (0.22 ~ 0.95) | 0.037 |

Notes: Model 1: unadjusted; Model 2: adjusted for sex, age; Model 3: additionally adjusted for marital status, smoking, drinking; Model 4: fully adjusted.

Abbreviations: OR, odds ratio; CI, confidence interval; HRR, hemoglobin-to-RDW ratio.

Table 5 Subgroup Analyses of HRR and Asthma Risk

| Group | NHANES Cohort | | | | Ganzhou Cohort | | | |
| --- | --- | --- | --- | --- | --- | --- | --- | --- |
| Variables | n (%) | OR (95%CI) | *P* | P for interaction | n (%) | OR (95%CI) | *P* | P for interaction |
| All patients | 19140 (100.00) | 0.85 (0.64 ~ 1.12) | 0.245 |  | 519 (100.00) | 0.21 (0.06 ~ 0.75) | 0.017 |  |
| Gender |  |  |  | 0.504 |  |  |  | 0.606 |
| Male | 9819 (51.30) | 0.89 (0.58 ~ 1.37) | 0.601 |  | 323 (62.24) | 0.18 (0.03 ~ 0.91) | 0.038 |  |
| Female | 9321 (48.70) | 0.75 (0.52 ~ 1.10) | 0.144 |  | 196 (37.76) | 0.36 (0.04 ~ 3.25) | 0.365 |  |
| Age |  |  |  | 0.103 |  |  |  | 0.909 |
| <60 | 12454 (65.07) | 0.72 (0.51 ~ 1.02) | 0.061 |  | 238 (45.86) | 0.22 (0.03 ~ 1.75) | 0.152 |  |
| ≥60 | 6686 (34.93) | 0.58 (0.35 ~ 0.97) | 0.039 |  | 281 (54.14) | 0.15 (0.03 ~ 0.85) | 0.032 |  |

Note: All models were fully adjusted.

Abbreviations: OR, odds ratio; CI, confidence interval; HRR, hemoglobin-to-RDW ratio.

Table 6 Vitamin D Mediation Analysis (NHANES Cohort)

| Path | Total Effect |  | Indirect Effect |  | Direct Effect |  | Proportion Mediated, %(95% CI) |
| --- | --- | --- | --- | --- | --- | --- | --- |
|  | Coefficient (95% CI) | P value | Coefficient (95% CI) | P value | Coefficient (95% CI) | P value |  |
| HRR→ Vitamin D → Asthma | -0.015(-0.054 ~ 0.023) | 0.44 | 0.011(0.003 ~ 0.007) | 0.001 | -0.026(-0.065 ~ 0.013) | 0.185 | 100% |

Note: Adjusted for sex, age, race, education, marital status, BMI, smoking, drinking, hypertension, diabetes.Abbreviations: Coef, coefficient; CI, confidence interval; HRR, hemoglobin-to-RDW ratio.
